# Service providers endorse integrated services model for youth with mental health and substance use challenges: findings from a discrete choice experiment

**DOI:** 10.1186/s12913-021-07038-3

**Published:** 2021-10-01

**Authors:** Lisa D. Hawke, Lehana Thabane, Srividya N. Iyer, Alexia Jaouich, Paula Reaume-Zimmer, Joanna Henderson

**Affiliations:** 1 Centre for Addiction and Mental Health, 80 Workman Way, Toronto, Ontario Canada; 2 University of Toronto Department of Psychiatry, 250 College Street, Toronto, Ontario Canada; 3 McMaster University, 1280 Main St W, Hamilton, Ontario Canada; 4 McGill University, 845 Sherbrooke St W, Montreal, Quebec, Canada; 5 Douglas Hospital Research Centre, 6875 Boulevard LaSalle, Montréal, Quebec, Canada; 6 Bluewater Health, 89 Norman St, Sarnia, Ontario Canada; 7 Canadian Mental Health Association Lambton Kent, 240 Grand Ave. West, Chatham, Ontario Canada

**Keywords:** Youth, Mental health, Substance use, Integrated services, Service provider

## Abstract

**Background:**

Given high rates of mental health and substance challenges among youth and substantial system access barriers, system innovation is required. Integrated youth services (IYS) models aim to transform youth mental health and substance use services by creating integrative, collaborative models of care in youth-friendly settings. This study examines service provider perspectives on the key service components to include in IYS models.

**Method:**

A discrete choice experiment modeled service provider preferences for the service components of IYSs. The sample includes 388 service provider/agency leader participants (age 18+) from youth-serving organizations in Ontario. Importance scores and utility values were calculated for 12 attributes represented by four levels each. Latent class analysis identified subgroups of participants with different preferences.

**Results:**

The majority of participants were direct service providers working in larger organizations in the mental health and/or substance use sectors in large urban centers. Participants strongly endorsed service models that provide rapid access to the widest variety of culturally sensitive service options, with supplementary e-health services, in youth-focused community settings with evening and weekend hours. They prefer caregiver involvement in youth services and treatment decisions and support youth and family engagement. Latent class analyses reveal three segments of service providers: a Youth-Focused Service Accessibility segment representing 62.1% (241/388) of participants, a Service Options segment representing 27.6% (107/388) of participants, and a Caregiver Integration segment representing 10.3% (40/388) of participants. Within these segments, the degree of prioritization of the various service components differ; however, the overall endorsement of the service components remains largely consistent across classes for most attributes. The segments did not differ based on demographic or agency characteristics.

**Conclusions:**

The core characteristics of IYS settings for youth with mental health and substance use challenges, i.e., rapid access to a wide range of youth-oriented services, are strong priorities of service providers and youth-serving agency leaders. These findings confirm that youth-oriented service providers endorse the importance and relevance of IYS models as a whole; strong service provider buy-in to the model is expected to facilitate development, implementation and scaling of IYS models. Hearing stakeholder perspectives, including those of service providers, youth, and caregivers, is essential to developing, effectively implementing, and scaling effective youth services.

**Supplementary Information:**

The online version contains supplementary material available at 10.1186/s12913-021-07038-3.

## Background

Adolescence and young adulthood is a critical period for the emergence of mental health and substance use challenges [1, 2]. Experiencing mental health or substance use challenges during the adolescent or emerging adult period is associated with multiple challenges in terms of immediate functioning and wellbeing, as well as long-term development and the transition to a productive adulthood [3, 4]. Recent epidemiological research shows that some 7.8% of Canadian youth have been diagnosed with a mood disorder, and 12.9% with an anxiety disorder [5]. Rates of diagnosed disorders among youth, self-reported dissatisfaction with their mental health, and service seeking, have been increasing over the past years [5]. Prescription opioid drug use for non-medical reasons is reported by 11% of students in grades 7 to 12 over the past year, while some 14% of youth in grades 9 to 12 report hazardous drinking and over 3% report signs of cannabis dependence [1].

Given the high rates of mental health and substance use challenges during this critical developmental period, access to high quality services is paramount. Unfortunately, the youth mental health service system is suboptimal. Long wait lists, complicated pathways to care, a lack of specialized services, stigma, and unresponsiveness constitute significant service access barriers [6–11]. Indeed, the majority of youth with mental health challenges do not access them [12]. Among those who do access services, dropout rates are high [13, 14], missing the opportunity to provide the support young people need to get back on track and show improvements that can have lifelong impacts.

To better address youth mental health needs, transformative system change is required. Reflecting this, a systems transformative movement is under way around the world, involving the creation of Integrated Youth Services (IYS) models of service delivery [15, 16]. The recently emerging IYS models aim explicitly to provide rapid access to effective services in a one-stop-shop model of care, where youth can access services for a wide range of health and psychosocial support needs in responsive, youth-friendly settings [17–20]. IYSs bring together service providers across a broad range of disciplines, such as psychotherapy, psychiatry, peer support, employment support, primary care, and other disciplines, who work in an integrated manner. Examples of IYS models emerging in Canada include the Foundry model in British Columbia, the pan-Canadian Access Open Minds model, and the Youth Wellness Hubs Ontario model [19–21].

Service integration in the Canadian youth mental health, substance use, and social services landscape has been inadequate in recent years, which leaves youth underserved in areas of care that are outside of the area of expertise of the organization they have accessed [22]. IYS models aim to remedy this gap in the system to provide the best available care for youth across a wide variety of areas of need. While recent work has identified the core components of IYS settings as a whole and around the world [15], it is not clear which components are most crucial to IYS service design relative to other components; this is an essential step in identifying which components should be prioritized when faced with limited resources and competing priorities.

The implementation science literature demonstrates the importance of taking into account stakeholder perspectives when implementing complex health interventions [23]. Service provider perspectives are particularly important, as interventions with strong service provider buy-in are more likely to be implemented effectively [24]. In the IYS model, it is particularly important to take service provider perspectives into account given the integration component, which brings together a wide variety of service providers from different backgrounds who may not be accustomed to working together in an integrated manner. Other stakeholder perspectives are also critical to building appropriate services. Notably, the perspectives of youth and the caregivers who support them must be taken into account to build services that are appealing to them, and are therefore more likely to be accessed by youth in need [25, 26].

This research project takes a rigorous approach to understanding the perspectives of multiple stakeholders in the development of IYS models of service delivery. Moving beyond the efficacy and effectiveness literature addressing specific treatments to be embedded into IYS settings, this study examines other diverse aspects of the setting that are not subject to efficacy trials, e.g., fundamental aspects of the setting such as the diversity of services, rapid access, hours of availability, and engagement. A scoping review [27] mapped various components of IYS settings around the world, and found that many components were similar across models, but some differed, such as the types of service providers; however, a lack of reporting on a number of aspects of care prevents drawing conclusions about the consistency of their implementation. To understand which components are the most important to implement in IYS settings, we used a discrete choice experiment (DCE) methodology to identify the relative importance of various setting characteristics as compared to others. We sought the perspective of service providers, youth, and caregivers on the key components of IYS services]. This paper presents the findings for the service provider sample; youth and caregiver perspectives are presented in companion manuscripts [28, 29].

*Research questions*. This study aimed to answer the following research questions: 1) What service characteristics do service providers consider the most important in building integrated youth services? 2) Do different segments of service providers exist, defined by different preferences? If so, what service characteristics and participant attributes define these segments?

## Method

The DCE is a quantitative approach that elicits consumer preferences regarding products or services with complex sets of hypothetical characteristics. DCE puts different characteristics (“attributes”) of a service or product head to head in hypothetical scenarios and asks the respondent to choose between them. The DCE makes it possible to determine the relative importance of different priorities over others. By combining multiple attributes represented by various levels of that attribute, it becomes possible to determine participants’ relative preference for one alternative over another in a complex set of service options. This methodology also identifies consumer subgroups with different sets of preferences. This DCE was designed following the International Society for Pharmacoeconomics and Outcomes Research taskforce’s report on Good Research Practices for Conjoint Analysis [30].

First, a scoping review was conducted to identify attributes and levels of possible services and service delivery options for an IYS [27]. Using this, draft attributes were created in consultation with the project team through iterative discussions, as well as opportunities to provide comments and rank their perspectives of the importance of items via project development surveys; these were programmed in REDCap [31, 32] software and distributed to the project team, made up of research team members (including community partners and youth) with substantial experience working in the IYS sphere. Feedback was used to progressively refine the attributes and levels. The resulting DCE survey was piloted with four youth, four caregivers, and two service providers in two locations, one large Canadian city and one rural area in Ontario, Canada.

**Fig. 1:**
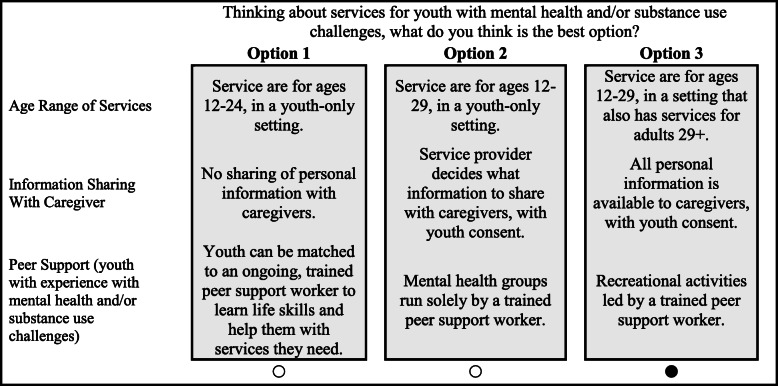
Sample DCE item

Feedback from each of these phases led to the finalization of the DCE, which contained 12 attributes of 4 levels each. The complete list of attributes and levels used in this study is presented in Table 1. The final DCE survey was programmed into Sawtooth Software’s SSI Web (version 9.8) and administered using that platform. The choice tasks utilized a partial profile design in which participants were asked to choose between three service options. Participants were asked to think about services for youth (aged 14–29) with mental health and/or substance use challenges and to select the best service option from among the three presented. The presented service options contained levels of three attributes; for a sample item, see Fig. 1. Surveys were balanced by an algorithm that optimized orthogonality and attribute/level balance. Each participant randomly received a different version of the survey.

**Table 1:** Complete attribute and level set used in the Discrete Choice Experiment

Attribute	Level 1	Level 2	Level 3	Level 4
1. Core Health Services	Only mental health counseling.	Mental health and substance misuse counseling.	Mental health and substance misuse counseling, and medication management.	Mental health and substance misuse counseling, medication management, and physical/sexual health.
2. Other Services	Education and employment services.	Housing, shelter and income support services.	Legal support services.	Choice of education, employment, housing, income support, and legal support services.
3. Caregiver Involvement	No caregiver involvement.	Caregivers receive own counseling.	Caregivers involved in family counseling with youth, with youth consent.	Caregivers involved in decisions regarding youth counseling, with youth consent.
4. Peer Support	Recreational activities led by trained peer support worker.	Can talk to a trained peer support worker, upon request.	Mental health groups run solely by a trained peer support worker.	Youth can be matched to an ongoing trained peer support worker to learn life skills and help them with services they need.
5. Cultural Sensitivity	Cultural background is not considered when picking a service or service provider.	Can ask for a service provider with a certain cultural background, when available.	Services are culturally sensitive and trauma-informed.	Culturally based services are available for cultures common in the local area.
6. E-Health Services	No e-health or electronic services.	Can schedule or reschedule appointments via email, text or online.	E-health services are offered 24/7 alongside in-person services during office hours.	All services are delivered only through a website, e-mail, text, or phone app.
7. Age Range	Services for ages 12–24, in a youth-only setting.	Services for ages 12–29, in a youth-only setting.	Services for ages 12–24, in a setting that also has services for children 0–12.	Services for ages 12–29, in a setting that also has services for adults 29 + .
8. Time of Appointments	Monday to Friday, 9 AM-5 PM.	Monday to Friday, 9 AM-9 PM.	Monday to Friday, 9 AM-9 PM, and Saturday, 9 AM-5 PM.	24/7.
9. Wait Times	See a counselor for the first time immediately, during office hours.	See a counselor for the first time after about 72 h.	See a counselor for the first time after about 1 month.	See a counselor for the first time after more than 1 month.
10. Location	Building or office in the community that specializes in mental health services.	Youth cafe and recreation centre.	Hospital or doctor’s office.	School setting.
11. Engagement	Youth and caregivers give feedback, e.g., anonymous surveys.	Youth and caregivers are on staff at the organization.	Youth and caregivers are on an advisory group that gives feedback on services and evaluation.	Youth and caregivers play a leadership role in making decisions for the organization.
12. Information Sharing	No sharing of personal information with caregivers.	All personal information is available to caregivers, with youth consent.	Service provider decides what information to share with caregivers, with youth consent.	Youth and service provider work together to decide what personal information to share with caregivers and how that can be helpful.

### Participants & procedure

Service provider participants were recruited from a comprehensive internal database of organizations offering services to Ontario youth with mental health and substance use challenges. Diverse perspectives were sought from service providers offering services in various sectors. Organizations were contacted by email with a link to the online survey and encouraged to disseminate that link to the service providers and organization leaders in their network. After completing informed consent, eligibility questions were presented; participants were deemed eligible if they either provided direct service to Ontario youth, or were in a leadership/management or administrative role for an agency that provided service to Ontario youth. An explanation of the DCE process was then provided, with a practice question prior to the DCE. Participants who completed the survey had the option of following a separate link to enter a draw to win one of a series of gift cards as a form of compensation; the draw entry was unlinked to the DCE survey and DCE responses were anonymous. The survey was conducted in English. Response time was a median of 15 min. The final sample size for the study was 388 participants. The study received approval from the Centre for Addiction and Mental Health Research Ethics Board.

### Other measures

In addition to the DCE, participants were asked demographic questions (e.g, age, gender, education, years of experience). They were also asked about their professional background, the primary and secondary sectors in which they offered care to youth, the age of the youth they served, and other descriptors of their roles.

### Data analysis

Descriptive statistics were calculated to describe the sample. The DCE was analyzed by estimating utilities for each participant using hierarchical Bayesian methods performed using Sawtooth Lighthouse Studio (9.8.1, [33]). Standardized, zero-centered utilities were used [34], with higher utility values reflecting the relative value of that level in relation to the other attribute levels; positive utility values represent a positive relative preference in relation to other attribute levels, while negative utility values reflect a negative relative preference. Each attribute’s relative importance was assessed by calculating the proportional utility of each of these attributes relative to the total utility provided by all attributes. Attributes that are selected more often by participants feature higher importance values.

Latent class analysis was employed to identify segments of participants with similar service preferences. Each participant was assigned a probability of belonging to a particular segment. Latent class analyses were performed using Latent Class module in Sawtooth Software. Each latent class solution was replicated five times at different starting seeds. Convergence was assumed when log-likelihood decreased by 0.01 or less. Based on the selected model, attribute rankings are presented descriptively. Chi-square tests were used to compare demographic characteristics across the segments. All other analyses were performed using SPSS 25 [35].

## Results

Participant characteristics are presented in Table 2. Participating service providers were highly likely to be female and be direct service clinicians (as opposed to in leadership or administrative positions). They were mostly from large urban centres, tended to have been their current position for five years or less, and were likely to have worked for large agencies with 30 or more employees. The majority provided services in the mental health and/or substance use sectors.

**Table 2:** Demographic and agency characteristics of service provider participants: *n* = 388

Demographic characteristic		*n (%)*
Age (years)	18–29	102 (27.3%)
	30–39	131 (35.0%)
	40–49	76 (20.3%)
	50+	65 (17.4%)
Gender	Male	48 (13.0%)
	Female	315 (85.4%)
	Another gender	6 (1.6%)
Highest education level	Bachelor’s degree or less	241 (62.9%)
	Graduate degree	142 (37.1%)
Years in current position	0–5	250 (64.8%)
	6–10	65 (16.8%)
	11–15	41 (10.6%)
	16+	30 (7.8%)
Years experience with youth mental health and/or substance use challenges	0–5	137 (36.8%)
	6–10	88 (23.7%)
	11–15	63 (16.9%)
	16–20	40 (10.8%)
	21+	44 (11.8%)
Current position at agency	Manager/administrator/executive director	89 (23.0%)
	Clinician/direct service staff	275 (71.1%)
	Other	23 (5.9%)
Agency characteristics		
Urban/rural region	Rural/small city/town	76 (19.6%)
	Medium urban	80 (20.6%)
	Large urban	228 (58.8%)
Agency size	≤ 30 employees	100 (26.6%)
	> 30 employees	276 (73.4%)
Primary service sector	Mental health	110 (28.4%)
	Mental health and substance use	110 (28.4%)
	Multi-service	63 (16.2%)
	Housing/shelter	26 (6.7%)
	Substance use	20 (5.2%)
	Education/vocational/employment	18 (4.6%)
	Child welfare/youth justice	15 (3.9%)
	Physical health	13 (3.4%)
	Other	13 (3.4%)
Age group served (years)	0–12	115 (29.6%)
	13–17	307 (79.1%)
	18–29	315 (81.2%)
	30+	177 (45.6%)
	Parents/caregivers/family	14 (3.6%)

The overall preferences for participants as a whole are presented in Table 3. Notably, participants positively endorsed offering the widest possible array of services, including within the ‘Core Health Services’ attribute, as well as supplementary services outside of the mental health and substance use spheres (‘Other Services’ attribute) and including trained peer support workers (‘Peer Support’ attribute). A rapid access model was also preferred (‘Wait Times’ attribute). They preferred offering culturally sensitive, trauma informed services (‘Cultural Sensitivity’) and providing supplementary e-health services and electronic scheduling (‘E-health Services’ attribute). Offering evening and weekend hours was positively endorsed, with some endorsement of 24/7 services (‘Time of Appointments’ attribute). Situating services in a community service setting or a youth café and recreation centre was preferred (‘Location’ attribute). Participants endorsed family counseling and involving caregivers in decisions (‘Caregiver Involvement’ attribute) as well as working with the youth to determine what information to share with caregivers (‘Information Sharing with Caregivers’). In addition, participants positively endorsed engaging youth and caregivers in an advisory group (‘Engagement’ attribute). For the ‘Age Range’ served attribute, the preferences were youth-focused services, but with low relative importance. The highest relative importances were for information sharing with caregivers, the variety of supplementary services, and supplemental e-health services.

**Table 3:** Mean zero-centered utility value (and standard error) for each level of each attribute, for the service provider sample as a whole

Attribute	Level	Mean Utility (Standard Error)
Core health services	Only mental health counseling	−73.66 (0.63)
	Mental health and substance misuse counseling	10.30 (0.69)
	Mental health and substance misuse counseling, and medication management	13.32 (0.51)
	Mental health and substance misuse counseling, medication management, and physical/sexual health	50.04 (0.52)
Other health services	Education and employment services	−23.57 (0.73)
	Housing, shelter and income support services	15.85 (0.56)
	Legal support services	−57.56 (0.80)
	Choice of education, employment, housing, income support, and legal support services	65.29 (1.07)
Peer support	Recreational activities led by a trained peer support worker	−11.52 (0.33)
	Youth can talk to trained peer support worker, upon request	−4.99 (0.17)
	Mental health groups run solely by a trained peer support worker	−18.20 (0.48)
	Youth can be matched to an ongoing trained peer support worker to learn life skills and help them with services they need	34.70 (0.28)
Wait times	See a counselor for the first time immediately, during office hours	56.47 (0.93)
	See a counselor for the first time after about 72 h	35.95 (0.22)
	See a counselor for the first time after about 1 month	−38.21 (0.53)
	See a counselor for the first time after more than 1 month	− 54.21 (0.21)
Cultural sensitivity	Cultural background is not considered when picking a service or service provider	−75.31 (1.04)
	Youth can ask for a service provider with a certain cultural background, when available	5.88 (0.25)
	Services are culturally sensitive and trauma informed	58.11 (0.87)
	Culturally-based services are available for cultures common in the local area	11.32 (0.16)
E-health services	No e-health services	−57.65 (0.30)
	Youth can schedule or reschedule appointments via email, text or online	37.76 (0.25)
	E-health services are offered 24/7 alongside in-person services during office hours	60.88 (0.88)
	All services are delivered only through a website, e-mail, text, or phone app	−40.99 (0.50)
Time of appointments	Monday to Friday, 9 AM-5 PM	−43.12 (0.95)
	Monday to Friday, 9 AM-9 PM	−1.28 (0.57)
	Monday to Friday, 9 AM-9 PM, and Saturday, 9 AM-5 PM	30.00 (0.62)
	24/7	14.40 (0.66)
Location	Building or office in the community that specializes in mental health services	42.27 (0.65)
	Youth café and recreation centre	43.71 (0.59)
	Hospital or doctor’s office	−59.40 (0.41)
	School setting	−26.58 (0.69)
Caregiver involvement	No caregiver involvement	−85.57 (2.09)
	Caregivers receive own counseling	18.28 (0.47)
	Caregivers are involved in family counseling with youth, with youth consent	45.20 (0.70)
	Caregivers are involved in decisions regarding youth counseling, with youth consent	22.09 (0.92)
Information sharing with caregivers	No sharing of personal information with caregivers	−65.50 (2.10)
	All personal information is available to caregivers, with youth consent	−1.99 (0.87)
	Service provider decides what information to share with caregivers, with youth consent	0.83 (1.02)
	Youth and service provider work together to decide what personal information to share with caregivers and how that can be helpful	66.67 (0.60)
Engagement	Youth and caregivers give feedback, e.g., anonymous surveys	−11.36 (0.35)
	Youth and caregivers are on staff at the organization	−19.27 (0.13)
	Youth and caregivers are on an advisory group that gives feedback on services and evaluation	25.45 (0.37)
	Youth and caregivers play a leadership role in making decisions for the organization	5.18 (0.37)
Age range	Services for ages 12–24, in a youth-only setting	7.69 (0.38)
	Services for ages 12–29, in a youth-only setting	12.32 (0.29)
	Services for ages 12–24, in a setting that also has services for children 0–12	−14.39 (0.46)
	Services for ages 12–29, in a setting that also has services for adults 29+	−5.62 (1.03)

Latent class analysis was conducted on the DCE items. Fit indices are presented in Table 4 for segment sizes of two to five. The three-segment model was selected based on a combination of fit, segment size, and interpretability. The attribute rankings for each of the three segments are presented in Table 5. Complete attribute and level results are presented in the Supplementary Figure.

**Table 4:** Fit indices for latent class solutions ranging from two to five classes

Number of classes	Log-likelihood	Percent certainty	AIC	CAIC	BIC	ABIC	Chi-Square
2	− 3939.65	28.08	8025.29	8573.84	8500.84	8268.87	3076.07
3	− 3876.77	29.23	7973.55	8800.13	8690.13	8340.59	3201.81
4	− 3832.52	30.03	7959.04	9063.65	8916.65	8449.54	3290.33
5	− 3788.09	30.85	7944.18	9326.82	9142.82	8558.14	3379.19

*AIC =* Akaike Information Criterion; *BIC =* Bayesian Information Criterion; *CAIC =* Consistent Akaike Information Criterion; *ABIC* = Sample size adjusted Bayesian Information Criterion

**Table 5:** Service provider attribute importance scores and rankings for core characteristics of integrated youth service hubs, by latent class segment

	Youth-focused service accessibility		Service options		Caregiver integration	
	I	R	I	R	I	R
Core Health Services	10.20	4	12.52	3	5.35	5
Other Services	8.70	7	16.01	1	5.22	6
Peer Support	5.39	10	3.84	11	4.03	10
Wait Times	10.75	2	7.18	6	7.62	4
Cultural Sensitivity	13.91	1	6.48	8	5.22	7
E-Health Services	10.48	3	7.64	5	14.00	3
Time of Appointments	8.31	8	3.66	12	3.21	11
Location	9.32	6	10.40	4	4.23	8
Caregiver Involvement	9.79	5	7.04	7	23.97	1
Information Sharing	7.59	9	14.35	2	21.10	2
Engagement	3.47	11	5.05	10	1.96	12
Age Range	2.08	12	5.85	9	4.11	9

R = Rank of each attribute’s importance score within informant and segment. I = Importance score of each attribute. Relative importance scores represent a percentage of value assigned to each attribute relative to the other attributes

### Segment 1: youth-focused service accessibility

The first segment, made up of 241 (62.1%) participants, endorsed services that meet the access needs of youth, leading to this segment being labelled Youth-Focused Service Accessibility. This segment strongly endorsed the Cultural Sensitivity, Wait Times, E-Health Services, and Core Health Services attributes. The attributes endorsed with the least relative priority levels were the age range served, youth and family engagement in the services, and peer support.

The Youth-Focused Service Accessibility segment prioritised services that were sensitive to the cultural and trauma background of youth. There was some endorsement of the options to have culturally-based services available for cultures common to the local area, or having the option to request a service provider with a certain cultural background. For the Core Health Services attribute, the level that provided mental health and substance misuse counseling, medication management, and physical/sexual health services received the highest utility value. Short Wait Times were also strongly preferred in this segment; Youth-Focused Service Accessibility participants felt that youth should be able to see a counselor either immediately, or after about 72 h. Finally, 24/7 E-Health Services that supplemented in-person services were strongly preferred, as was the option to schedule or reschedule appointments via email, text, or online.

### Segment 2: service options

Segment 2, which is comprised of 107 (27.6%) participants, was labelled the Service Options segment. The decisions of these participants were driven by service-related attributes, including Core Health Services and Other Services. They also prioritized the Information Sharing and Location attributes. The attributes endorsed with the lowest level of relative priority were the time of appointments, peer support, and youth and family engagement in the organization.

The Service Options segment selected levels representing the most diverse services, including mental health and substance misuse counseling, medication management, and physical/sexual health services from the Core Health Services attribute. This segment also valued the more ancillary options presented in the Other Services attributes, such as education, employment, housing, income support, and legal support. Levels that were limited to employment/education or legal support services both were negatively endorsed. In the Information Sharing attribute, this segment preferred to work with youth to decide which information to share with caregivers. There was little endorsement, positive or negative, for the levels in which the service provider decides with information to share with caregiver, or in which information is available to caregivers with youth consent. There was a negative utility value for no information sharing with caregivers. For the Location attribute, this segment preferred that services be delivered in a dedicated mental health setting or in a casual location such as a café or recreation centre, as opposed to a hospital or school setting.

### Segment 3: caregiver integration

Segment 3, comprised of 40 (10.3%) participants, consists of participants who preferred the attributes Caregiver Involvement, Information Sharing, E-Health Services, and Wait Times. This segment was labelled the Caregiver Integration segment. The attributes reflecting the least relative priority were youth and family engagement, the time of appointments, and peer support.

Service providers in the Caregiver Integration segment endorsed a high level of Caregiver Involvement, with caregivers involved in decisions regarding youth counseling, participating in family counseling, or receiving separate counseling from youth. Caregivers receiving their own, separate counseling was less important, although still positively endorsed, while the level specifying no caregiver involvement received a large, negative utility value from this segment. This segment also preferred information sharing with caregivers. This included positive utility values for levels specifying that youth and service providers would work together to decide what information to share, that service providers decided what information to share, and that information is available to caregivers with youth consent. The level specifying that there would be no sharing of personal information with caregivers received negative utility values. The Caregiver Integration segment believed that e-health services should be offered as a supplement to in-person service, and that service users should be able to schedule or reschedule appointments via email, text, or online. Finally, for the Wait Times attribute, service providers preferred rapid access.

Chi-square analyses of demographic and agency characteristic variables indicated that the segments did not differ based on any of these factors (Table 6).

**Table 6:** Demographic and agency characteristics of service provider participants by latent class segment

		Youth-focused service accessibility *n* = 241	Service options *n* = 107	Caregiver integration *n* = 40		
		*n (%)*	*n (%)*	*n (%)*	*p*	*Cramer’s V*
Age (years)	18–39	142 (60.4%)	65 (65.7%)	26 (65.0%)	.622	.05
	40+	93 (39.6%)	34 (34.3%)	14 (35.0%)		
Gender^1^	Male	26 (11.6%)	15 (15.2%)	7 (17.9%)	.444	.07
	Female	199 (88.4%)	84 (84.8%)	32 (82.1%)		
Highest education level	Bachelor’s degree or less	147 (61.8%)	69 (65.1%)	25 (64.1%)	.829	.03
	Graduate degree	91 (38.2%)	37 (34.9%)	14 (35.9%)		
Years in current position	0–5	162 (67.2%)	66 (62.3%)	22 (56.4%)	.346	.07
	6+	79 (32.8%)	40 (37.7%)	17 (43.6%)		
Years experience with youth mental health and/or substance use challenges	0–5	78 (33.2%)	43 (43.4%)	16 (42.1%)	.161	.10
	6+	157 (66.8%)	56 (56.6%)	22 (57.9%)		
Current position at agency	Program manager/administrative/ organizational leadership	56 (25.0%)	23 (22.5%)	10 (26.3%)	.857	.03
	Clinical/direct staff	168 (75.0%)	79 (77.5%)	28 (73.7%)		
Agency characteristics						
Urban/rural region	Non-large urban	90 (37.7%)	48 (45.7%)	18 (45.0%)	.314	.08
	Large urban	149 (62.3%)	57 (54.3%)	22 (55.0%)		
Agency size	≤ 30 employees	59 (25.5%)	32 (30.2%)	9 (23.1%)	.583	.05
	> 30 employees	172 (74.5%)	74 (69.8%)	30 (76.9%)		
Primary service sector	Mental health/substance use	154 (63.9%)	66 (61.7%)	29 (72.5%)	.672	.06
	Multi-service agency	42 (17.4%)	17 (15.9%)	4 (10.0%)		
	Other agencies	45 (18.7%)	24 (22.4%)	7 (17.5%)		
Age group served (years)	0–12	73 (30.3%)	28 (26.2%)	14 (35.0%)	.544	.06
	13–17	197 (81.7%)	79 (73.8%)	31 (77.5%)	.237	.09
	18–29	197 (81.7%)	83 (77.6%)	35 (87.5%)	.366	.07

Additional genders were removed from the analyses due to small cell sizes

## Discussion

This study aimed to understand the perspectives of service-providing stakeholders on the service characteristics most important to include in IYS settings for youth mental health and substance use disorders. Results revealed that participants strongly endorse service models that provide rapid access to the widest variety of culturally sensitive service options, with supplementary e-health services, in youth-focused community settings with evening and weekend hours. They prefer caregiver involvement in youth services and treatment decisions, and support youth and family engagement. There were three segments of service providers/organization leaders with somewhat different perspectives. The largest segment most strongly preferred youth-oriented accessibility factors. Another segment, representing over a quarter of service providers, emphasized the importance of offering the most diverse possible set of services to meet a wide range of youth needs. A minority of participants fell into a group that valued the involvement of the youth’s caregiver as a top relative priority. The segments did not differ based on demographic characteristics.

Obtaining the buy-in of service providers, service providing agencies, and other stakeholders is essential to the successful implementation of complex health interventions [36, 37]. Referred to as part of the “inner setting” of the implementation environment in the implementation science literature, service providers and agencies must have positive attitudes toward an intervention and believe it represents an improvement in order to implement an intervention with fidelity [36, 37]. A IYS implementation study found that a strong inner setting during the implementation process was important for the successful implementation of the intervention [38]. The current study further demonstrates that service providers and agency leaders across a sample representing diverse service organizations strongly value youth-focused core service components that are foundational to IYS settings, such as rapid access to a variety of health services for a wide range of youth needs. While the degree of prioritization of different service components differed somewhat across latent segments, overall preferences were very similar for most components of care and service providers universally positively endorsed key preferences that define IYS models. IYS models of youth services, then, appear to be a strong fit with the preferences of service providers at youth-serving agencies in the current youth service landscape, in which IYS services are in the early stages of being scaled up, but are not yet universal.

Caregivers of youth have expressed the need to be more involved in their youth’s treatment. Service providers as a whole also support caregiver involvement in care. Information sharing with caregivers is a top priority for service providers, while they also endorse involving caregivers in counseling and decision-making. Indeed, caregivers can provide the impetus for youth to seek services and many wish to be closely involved in their care [8, 39–41]. It is important for service providers in IYS settings to recognize this and negotiate with the youth and caregiver to ensure that the caregivers are involved in an appropriate manner.

IYS models of service delivery are rapidly expanding around the world, including multiple Canadian models [21]. Reviews have described prevalent characteristics of such models and presented evidence of positive outcomes [15, 16]. IYS models strive to integrate many service components and characteristics considered to be youth friendly and consistently define themselves as youth-friendly settings [9]. The goal is to make these service settings as comprehensive as possible, including a broad range of services to meet youth needs across mental health and substance use, but also including attention to important factors such as employment, education, housing, and other social determinants of health. Indeed, given the preferences expressed by service providers, all of these components of care and care settings should continue to be a driving objective of service development. Based on the current findings, youth service developers and funders of such services should prioritize the integration a wide variety of services, including e-health service components, while ensuring rapid access and developing strong policies around caregiver involvement. In doing so, they will optimize service provider and agency buy-in to their models by building services that reflect these key stakeholder preferences. Youth and caregiver preferences should also be taken into account when identifying priorities for IYS service development [28, 29].

### Strengths and limitations

The DCE methodology makes it possible to identify and model stakeholder perspectives in a rigorous manner, with a particular strength of identifying relative priorities for certain service characteristics over others. These results can guide system designers as they engage in difficult decision-making processes, when faced with competing priorities and limited resources that require them to focus on the most important service characteristics first. Areas of agreement across segments highlight the highest priority area for service development, while areas of disagreement represent areas of possible design flexibility, as well as focus areas for consensus building to be contextualized based on service user preferences.

The study was conducted throughout Ontario, Canada, and reached participants across a diversity of youth service sectors. The results are therefore considered to illustrate broad themes within youth-serving agencies of various kinds across Ontario. However, the recruitment process was limited to one Canadian province and the sample may not have been representative of Ontario youth-serving organizations. Results may therefore not be generalizable outside of Ontario or in organizations with characteristics not represented in the sample. Different subgroups of participants may emerge with a more diverse sample. The current results refer to service provider/organizational leadership preferences only and should be interpreted together with the perspectives of service users [28, 29]. It is also important to keep in mind that a DCE item set is not a psychometrically validated assessment tool. The items were developed through a substantial, collaborative process in which stakeholders were engaged; however, it is possible that some priorities of participants were not included in the item set and overlap between items may have affected the relative importance attributed to different attributes and levels. This study does not make it possible to identify the efficacy or effectiveness of any of the service components. Given the complex nature of IYS settings and service pathways, future effectiveness research should examine outcomes of youth within the model as a whole, to supplement outcome research on individual components of care. Future research should also consider effectiveness based on the service components that have been implemented in a given IYS model.

## Conclusions

The core characteristics of IYS settings, i.e., rapid access to a wide range of youth-oriented services, are considered strong priorities by service providers and youth-serving organizational leaders. These findings confirm that youth-oriented service providers endorse the importance and relevance of the IYS model as a whole. The development, implementation and scale of IYS models will be facilitated by service provider buy-in to the model. Future IYS research and development work should take service provider priorities into account to build the most responsive, comprehensive service models possible to address youth service access barriers and substantially improve the youth mental health, substance use, and wellness support sector. In building such models, hearing the preferences of diverse stakeholders, including service providers, youth, and caregivers, is essential.

## Supplementary Information

**Additional file 1.**

## Data Availability

The datasets used and/or analysed during the current study are available from the corresponding author on reasonable request and upon Research Ethics Board approval.

## Abbreviations

IYS: Integrated Youth Services
DCE: Discrete Choice Experiment
