# Supplementary material for: Service providers endorse integrated services model for youth with mental health and substance use challenges: findings from a discrete choice experiment
Source: BMC Health Serv Res. 2021 Oct 1;21:1035. doi: 10.1186/s12913-021-07038-3 (PMC8487137; doi:10.1186/s12913-021-07038-3)
Supplement: Supplementary file 1 — Additional file 1. [file 12913_2021_7038_MOESM1_ESM.docx]

**Supplemental Material:** Level endorsements for each attribute for the three service provider segments

| Legend |  |  |  |
| --- | --- | --- | --- |
|  | Youth-Focused Service Accessibility (n = 241) | | |
|  | Service Options (n = 107) | | |
|  | Caregiver Integration (n = 40) | | |

**Figure 1.** Level endorsements for the Core Health Services attribute

**Figure 2.** Level endorsements for the Other Health Services attribute

**Figure 3.** Level endorsements for the Peer Support attribute

**Figure 4.** Level endorsements for the Wait Times attribute

**Figure 5.** Level endorsements for the Cultural Sensitivity attribute

**Figure 6.** Level endorsements for the E-Health Services attribute

**Figure 7.** Level endorsements for the Time of Appointments attribute

**Figure 8.** Level endorsements for the Location attribute

**Figure 9.** Level endorsements for the Caregiver Involvement attribute

**Figure 10.** Level endorsements for the Information Sharing with Caregivers attribute

**Figure 11.** Level endorsements for the Engagement attribute

**Figure 12.** Level endorsements for the Age Range of Services attribute
